# Patterns and temporal change of psychopathological symptoms among inpatients with alcohol use disorder undergoing a twelve-step based treatment

**DOI:** 10.1016/j.abrep.2020.100302

**Published:** 2020-09-08

**Authors:** Zsolt Horváth, Mariann Tremkó, Zsolt Fazekas, András Tóth, Zsolt Petke, Judit Farkas, Mark D. Griffiths, Zsolt Demetrovics, Róbert Urbán

**Affiliations:** a Institute of Psychology, ELTE Eötvös Loránd University, Izabella utca 46, Budapest H-1064, Hungary; b Doctoral School of Psychology, ELTE Eötvös Loránd University, Izabella utca 46, Budapest H-1064, Hungary; c Department of Addictology, Nyírő Gyula National Institute of Psychiatry and Addictions, Lehel utca 59-61, Budapest H-1135, Hungary; d Psychology Department, Nottingham Trent University, Nottingham, United Kingdom

**Keywords:** Alcohol Use Disorder (AUD), Psychopathological symptoms, Twelve-step based treatment, Minnesota model, Alcohol comorbidity

## Abstract

**Background:**

Patients diagnosed with Alcohol Use Disorder (AUD) present an increased risk for experiencing severe internalizing and externalizing symptoms. Involvement in twelve-step based treatment programs, such as the Minnesota Model (MM), can contribute to improvement of an individual’s psychopathological symptom profile. The present study’s main objective was to examine profiles and change trajectories of psychopathological symptoms of AUD subgroups during an eight-week long period of MM treatment attendance.

**Method:**

Inpatients with AUD (N = 303) who attended MM treatment programs participated in the present study. Latent Class Growth Analysis (LCGA) was used to evaluate the psychopathological symptom change trajectories assessed by using the Brief Symptom Inventory (BSI). Multiple comparisons and multinomial logistic regression were performed to validate the subgroups.

**Results:**

Three subgroups were identified: low severity (48.5%), moderate severity (35.2%), and high severity (16.2%) symptomatic subgroups. The moderate severity class demonstrated the largest effect in terms of symptoms decrease. Higher severity classes showed significantly higher rates of harmful alcohol drinking and drinking motives.

**Conclusions:**

The present study identified three severity-based subgroups which indicate that psychopathology sits on a spectrum of severity among AUD patients. The findings highlight the associations between AUD and internalizing symptoms, negative reinforcement drinking motives, and the symptomatic improvement that can occur among those participating in MM treatment programs.

## Highlights

• Psychopathological symptom profiles and trajectories were examined among AUD inpatients.
• Three quantitatively different subgroups were identified in terms of psychopathological symptoms.
• Classes were discriminated by different psychopathological symptom change trajectories.
• Subgroups with more severe psychopathological symptoms used alcohol in a more harmful way.
• Drinking of the more severely affected classes were more motivated by coping and conformity motives.

## 1 Introduction

Alcohol use disorder (AUD) is a chronic problem causing significant psychological, physical, interpersonal, and social burden among alcohol users and their environment. Extensive empirical research suggests that AUD frequently co-occurs with diverse or even multiple forms of psychiatric disorders, such as major depressive disorder, panic disorder, obsessive-compulsive disorder, generalized anxiety disorder, post-traumatic stress disorder, social phobia, or antisocial personality disorder (for review, see Bradizza, Stasiewicz, & Paas, 2006). Comorbid externalizing and internalizing psychopathology are often associated with more severe subtypes of AUD in terms of clinical characteristics and prognosis (e.g., higher drinking severity, worse health status; Hildebrandt et al., 2017, Moss et al., 2010) and harmful treatment-related consequences (e.g., higher level of treatment drop-out, increased vulnerability of early and long-term relapse after treatment; Farren and McElroy, 2010, Krawczyk et al., 2017). Furthermore, some psychopathological symptoms (e.g., depression or anxiety symptoms) have an integral role in the pathology of AUD, such as progression into more pathological stages of AUD, motivation and maintenance of compulsive alcohol use, craving and relapses, or even during a period of long-term abstinence. For example, the allostatic model assumes that during the progression from early stages (e.g., preoccupation with alcohol use, frequent intoxication) to the more severe, compulsive stage of AUD, there is a shift in the motivational background of alcohol use from positive reinforcement (i.e., drinking to facilitate positive emotions and hedonic states) to negative reinforcement (i.e., drinking to alleviate negative affective states related to withdrawal), and function of negative affectivity becomes central due to adverse modifications in the reciprocal emotion-regulation and reward-regulation systems (Koob, 2013, Le Moal and Koob, 2007). Another possible form of comorbidity is alcohol-induced mental disorders, such as mood, anxiety, bipolar and psychotic disorders, where psychopathological symptoms last for 1–6 months following excessive use of alcohol (Saunders, 2017).

Changes in psychopathological symptoms over time among individuals with AUD have been examined in a large body of existing literature. For example, studies using meta-analysis and systematic review have reported that patients with comorbid AUD and psychiatric disorders show improvements in depression, anxiety, and post-traumatic stress disorder symptoms due to involvement in antidepressant pharmacological therapies, cognitive behavioral therapies, and motivational interviewing (Baker et al., 2012, Foulds et al., 2015, Hobbs et al., 2011, Riper et al., 2014, Roberts et al., 2015). Moreover, previous studies have demonstrated that participation in structured twelve-step based therapeutic approaches, such as the Minnesota Model (MM) and the Twelve Step Facilitation (TSF) treatment, not only have beneficial impact on drinking-related outcomes (e.g., reaching longer periods of abstinence; Grønbaek and Nielsen, 2007, Kelly et al., 2020, Project Match Research Group, 1998), but also related to improvements in psychopathological-related (e.g., attenuation of depression symptoms) outcomes (Andó et al., 2016, Worley et al., 2012).

In addition to other analytical approaches (e.g., factor analysis; Harford, Yi, Chen, & Grant, 2015), one possible way to examine structure and patterns of psychopathological comorbidity among individuals with AUD is to use person-centered analyses, such as latent class or profile analysis (Urbanoski, Kenaszchuk, Veldhuizen, & Rush, 2015). These methods allow to obtain better understanding on the heterogeneity within AUD by distinguishing subgroups of individuals where members within each identified class show similar profiles and combinations of psychopathological comorbidity. Among individuals with AUD either from treatment seeking or general population samples, previous studies have identified three to five distinct subgroups based on indicator variables assessing co-occurring psychopathological symptom levels or disorder presence (Glass et al., 2014, Müller et al., 2019, Urbanoski et al., 2015, Villalobos-Gallegos et al., 2017, Wallen et al., 2019). These studies have been conflicting in terms of the observed differences between the identified latent classes: quantitative (e.g., parallel and severity-based psychopathological symptom profiles; Villalobos-Gallegos et al., 2017) and qualitative differences have been suggested between the psychopathology-based subgroups (e.g., classes with mainly externalizing and internalizing comorbidity; Glass et al., 2014).

However, to the best of the authors’ knowledge, no previous study has examined how latent classes of AUD change over time or due to treatment attendance in terms of co-occurring psychopathological symptom levels. Investigation of temporal change patterns of psychopathology-based latent classes might contribute to broaden existing knowledge about AUD in two broad aspects. First, it would be possible to obtain more detailed understanding on the structure of comorbid psychopathology among individuals with AUD. Considering the principles of latent class growth analysis (Jung & Wickrama, 2008), it is assumed that individuals with AUD might show substantial variability how their psychopathological symptom levels change over time and different subtypes of AUD might present various trajectories. Therefore, comorbid psychopathology-based subgroups might have heterogenous characteristics not only in terms of severity (e.g., mild level vs. high level of psychopathology) and qualitative aspects (e.g., presence of comorbid internalizing vs. externalizing psychopathology) of psychopathology, but in temporal patterns as well (e.g., higher degree of change vs. resistance of change in the level of psychopathology). Based on this approach, clinical prognosis of subgroups of AUD can be assessed in terms of comorbid psychopathology (Urbanoski et al., 2015).

Second, by identifying comorbid psychopathology-based latent classes it is possible to examine how these subgroups of AUD might differ in terms of treatment response (Lanza & Rhoades, 2013). Existing literature data has suggested that treatment effectiveness of a given intervention can vary as a function of membership of latent classes which are characterized with different profiles of alcohol misuse and internalizing and externalizing psychopathology (Roos, Bowen, & Witkiewitz, 2017). However, to the best of the authors’ knowledge, no previous study has examined treatment-seeking individuals with AUD profile characteristics as well as temporal change patterns of comorbid psychopathology-based latent classes during an AUD-related treatment participation. Classification participants based on psychopathological symptom severity and change profiles can help (i) to identify subgroups of AUD to whom a given treatment form can be considered as more effective, (ii) to design more individually-customized interventions and (iii) to specify in a more in-depth way how comorbid psychopathological symptoms alter treatment outcomes (Lanza and Rhoades, 2013, Urbanoski et al., 2015, Villalobos-Gallegos et al., 2017).

The aim of the present study was to examine patterns of severity and changes of psychopathological symptoms among subgroups of AUD inpatients attending a twelve-step based treatment program. It was hypothesized that the psychopathological-based latent classes of AUD can be discriminated in terms of symptom severity and temporal change patterns (Jung and Wickrama, 2008, Villalobos-Gallegos et al., 2017). However, it is important to note, that theoretical and practical conclusions, such as structure of psychopathology in the AUD population or assessment of specific treatment effects, can only cautiously be drawn from the present study due to its methodological limitations (e.g., lack of randomized controlled trial, follow-up data collection, examination of treatment moderators and mediators, potential self-selection bias of the participants, and inclusion of relevant confounding variables).

## 2 Methods

### 2.1 Participants and procedure

The present study was conducted between 2013 and 2018 at the Nyírő Gyula National Institute of Psychiatry and Addictions, Budapest, Hungary. The study specifically focused on the MM treatment program which was primarily designed for patients with AUD or gambling problems. The treatment includes eight weeks of community-based residential care which harmonizes professional treatment approaches and principles of the twelve-step based self-help group of Alcoholics Anonymous (AA). In line with the concepts of AA, the treatment primarily aims to aid patients to reach and maintain long-term abstinence. The treatment program emphasizes relevance of group-therapeutic context, community-based factors, and therapeutic effect of ‘here-and-now’ in AUD-related and psychological progress. Recovery of patients is followed and guided by a multidisciplinary staff team comprising professional therapeutic specialists (i.e., clinical psychologist, psychiatrist, addictology-specific consultant), nurses and recovering helpers (i.e., individuals who have successfully recovered from addiction-specific problems and who provide counselling in an addiction-specific treatment program; Doukas & Cullen, 2010). During the treatment, various group and individual psychotherapeutic techniques are applied, including daily AA meetings, specific group meetings based on the theoretical and practical principles of AA, assertiveness training, relaxation and stress management training, teaching of effective coping and relapse prevention skills, art therapeutic sessions, group meetings for affected family members, and psycho-education. The structure of the applied therapeutic techniques in the treatment program is presented in Table 1, while short description of the main treatment forms is shown in Table 2. Each patient has an individual consultant from the staff team, therefore frequent and regular individual consultations also support and facilitate progression of the participants. The daily schedule of the therapeutic sessions and related assignments are controlled by timetables (see: Table 1) in addition to the predefined, eight-week long structure for the program. The first two weeks of the treatment are relatively restrictive (e.g., it is not allowed to leave the therapeutic site or to have visitors) which aims to gain a more self- and therapeutic-focused attention, while in subsequent weeks, amongst others, participation in daily AA meetings and twelve-step-related functions (e.g., selection of a sponsor) are emphasized to a greater degree. An adaptation period is held in the seventh week of the treatment. During this time, the participants stay at their home, which, for example, allow for them to monitor their experiences in their ordinary environment (for further details, see Tóth, 2018). Although the main therapeutic approaches and characteristics of the treatment program remained unchanged from the beginning (e.g., structured organization and timetable, main therapeutic sessions, requirements for enrollment), it is important to take into account that some changes might have affected treatment processes over time (e.g., development of therapeutic skills and expertise over time, changes in the staff).

**Table 1:** General timetable for the treatment program.

Time	Monday	Tuesday	Wednesday	Thursday	Friday	Saturday	Sunday
7:30–8:00	Morning thoughts	Morning thoughts	Morning thoughts	Morning thoughts	Morning thoughts	Morning thoughts	Morning thoughts

8:00–8:30	–	–	–	–	–	–	–

8:30–9:00	Just for today	Just for today	Just for today	Just for today	Just for today	Breakfast

9:00–9:30	Breakfast	Breakfast	Breakfast	Breakfast	Breakfast	Breakfast	Film group with discussion

9:30–10:00	Assertiveness training, or AA-steps group, or Psychoeducation	Assertiveness training, or AA-steps group, or Psychoeducation	Assertiveness training, or AA-steps group, or Psychoeducation	Art therapeutic group	Week ending group	–	

10:00–10:30	Retrospective meeting for the week			

10:30–11:00	Week starting group	–	–	Film group with discussion

11:00–11:30	Assertiveness training, or AA-steps group, or Psychoeducation	Full department group	–		–	–

11:30–12:00	–

12:00–12:30	Lunch	Lunch	Lunch	Lunch	Lunch	Lunch	Lunch

12:30–13:00	Film group with discussion

13:00–13:30		Individual consultations and/or working on therapeutic tasks	Individual consultations and/or working on therapeutic tasks	Individual consultations and/or working on therapeutic tasks	Group for affected family members	Emotion-focused board game	–

13:30–14:00							

14:00–14.30	–						

14:30–15:00		–					

15:00–15:30			Working on therapeutic tasks	Stress management training	Stress management training	–

15:30–16:00						

16:00–16:30	–		–	–

16:30–17:00		

17:00–17:30	AA meeting		AA meeting	AA meeting	AA meeting	AA meeting	AA meeting

17:30–18:00							

18:00–18:30							

18:30–19:00

19:00–19:30

19:30–20:00

20:00–20:30	Dinner	Dinner	Dinner	Dinner	Dinner	Dinner	Dinner

20:30–21:00	Evening thoughts	Evening thoughts	Evening thoughts	Evening thoughts	Evening thoughts	Evening thoughts	Welcome back meeting

**Table 2:** Main therapeutic forms of the treatment program.

Name of the session	Short description and examples
Morning thoughts	Group meeting which is guided by a trained nurse. Example session: a daily quote is selected, and participants are asked to think about how it can be related to their recovery.
Evening thoughts	Group meeting which is guided by a trained nurse. Example session: participants are asked to summarize and describe their day, daily emotions, etc.
Just for today	AA-specific group meeting which is guided by a recovering helper. The session follows theoretical and practical approaches of the AA. Example session: discussion of the experiences, questions of the participants regarding daily AA meetings and their process of recovery.
Assertiveness training	Relapse prevention-specific group session which is guided by a clinical psychologist. Example session: practicing how to handle and cope with situations when there is a risk for substance use.
AA-steps group	AA-specific group session which is guided by a recovering helper. It focuses on the first two steps of the AA. It is held between the third and sixth weeks.
Psychoeducation	Group meeting which is guided by a psychiatrist with the aim of facilitating knowledge about substance use disorder-related mechanisms. Example session: discussion of the main characteristics of substance use disorders, education about relevant psychological defense mechanisms.
Week starting group	Group meeting where all inpatients and staff team members are included. Community-based therapeutic approaches are applied in this group. Themes of the sessions are not pre-defined, and are typically determined by the inpatients. Example session: discussion of problems occurring during the weekend with family members.
Week ending group	Group meeting where all inpatients and staff team members are included. Community-based therapeutic approaches are applied in this group. Themes of the sessions are not pre-defined, and are typically determined by the inpatients. Example session: preparation for returning home and meeting with family members during the weekend.
Full department meeting	Group meeting where all inpatients and staff team members of the Department of Addictology are involved (i.e., not just from the Minnesota treatment program). Example session: discussing issues which affect therapeutic work of the Department of Addictology.
Art therapeutic group	Creative group meeting which is held by a clinical psychologist. Example session: participants are encouraged to present a given problematic psychological aspect of their life by drawing.
Film group with discussion	Participants watch films which are relevant in terms of substance misuse and recovery, which is either followed by a group discussion or participants are asked to summarize their thoughts and feelings, emotions about the movie in written form.
Individual consultations	Each patient has an individual consultant from the staff team, therefore frequent and regular individual consultations also support and facilitate progression of the participants.
Working on therapeutic tasks	Some therapeutic forms require participants to prepare therapeutic tasks or homework to facilitate progression. Example tasks: writing an autobiography, reading the Big Book of AA and about the twelve steps.
Stress management training	Group meeting which is held by a clinical psychologist. Relaxation and imaginative elements are included in this therapeutic form. Example session: teaching and practicing basic elements of autogenic training.
Group for affected family members	Group meeting where affected family members of the inpatients are included. The meetings’ focus is not on the patient but on providing support and opportunity for consultations for family members. Example session: discussing how the affected family members trying to cope with the individual showing problematic alcohol use.
AA meeting	Patients are required to participate in AA meetings on a daily basis. This therapeutic form is held outside of the treatment site.
Therapeutic forms on Saturday and Sunday	Patients are only required to stay on the treatment site on the first weekend of the treatment program. Therefore, the treatment forms presented in Table 1 on these days are not relevant on other weeks of treatment program.

Overall, 303 inpatients (180 males and 123 females) with AUD participated in the present study. A total of 218 participants (71.95%) successfully completed the eight-week long program, while 85 inpatients (28.05%) dropped out from the treatment before completion (see ‘Sample characteristics’ subsection). Every patient who were admitted to the treatment program between March 2013 and April 2018 were included as a participant in the present study. Each of the attending patients agreed (and provided informed consent) to participate in the study. Detoxification was undertaken prior to the participants’ enrolment in the program as it was required from them to have at least one-week long abstinence and show absence of physical and acute psychological withdrawal before starting the treatment program. An approximately one-hour long, semi-structured interview was administered by the treatment staff before treatment enrollment. The interview took place at the treatment site, and an addition to the individual with AUD, one of his/her relatives also participated in the interview. The presence of the affected family members provides the opportunity to obtain a more accurate picture about the complex nature of problematic alcohol use not only for the staff but also for the individual with AUD (e.g., by asking them to share how the problematic alcohol use affected the function of the family). It also helps in observing possible psychological dynamics within the family, and motivates the relatives to participate in the treatment program (i.e., the group of affected family members). During the interview motivation for treatment and change in the problematic use of alcohol, as well as aspects of treatment contraindication, were evaluated. Lack of organic dementia, severe personality disorder, tendency to act out, and acute suicide risk were prerequisites for treatment involvement. Moreover, data were collected during the interview concerning socio-demographics (e.g., age, gender, education, and work history), sources of family or social support, while psychiatric anamnesis was also evaluated (e.g., family history of substance misuse, previous suicide attempts, psychiatric-, AUD- or SUD-related treatment involvement history). The present study also assessed if a participant had received some forms of psychiatric-related or AUD-related pre-care shortly before the treatment program. Pre-care involvement was considered if a participant was directed from an inpatient psychiatric- or AUD-related department to the MM treatment program, or reported a participation in a psychiatric-, AUD- or SUD-related treatment program within one month before the start of the MM program. Standardized questionnaires were used at two measurement points. On first entering the treatment program, alcohol consumption (e.g., harmful alcohol consumption, drinking motives), and psychopathological-related aspects were assessed. At the end of the treatment program, the levels of psychopathological symptoms were re-assessed. Research assessment was conducted by the treatment staff at both measurement points. Among those participants who dropped out from the treatment before completion, data were only available at the first measurement point. Systematic follow-up data collection either after successful treatment completion or treatment interruption was not carried out.

### 2.2 Measures

#### 2.2.1 Alcohol Use Disorders Identification Test (AUDIT)

The 10-item AUDIT was used to assess the degree of harmful alcohol consumption and consequences (Saunders, Aasland, Babor, De La Fuente, & Grant, 1993; Hungarian version: Gerevich, Bácskai, & Rózsa, 2006). Participants were assessed with the instrument before the beginning of the treatment program. In line with the assumed unidimensional structure of the scale (Skogen, Thørrisen, Olsen, Hesse, & Aas, 2019), total scale point was used for analyses. The scale had sufficient internal consistency in the present sample (Cronbach’s α = 0.72).

#### 2.2.2 Brief Symptom Inventory (BSI)

The present study assessed psychopathological symptom severity using the 53-item BSI at the beginning and at the end of the treatment program (Derogatis & Savitz, 2000; Hungarian version: Unoka et al., 2004). Previous research findings supported that the BSI was an appropriate instrument to reflect on the hierarchical structure of psychiatric symptoms by assessing general and specific factors of psychopathology simultaneously (Urbán et al., 2014), therefore general symptom severity, anxiety, depression, hostility, interpersonal sensitivity, obsessive-compulsive, paranoid ideation, phobic anxiety, psychoticism, and somatization scale scores were considered for analyses. The subscales of the BSI displayed acceptable levels of internal consistency at both measurement points (pre-treatment: Cronbach’s α = 0.73–0.89; post-treatment: Cronbach’s α = 0.68–0.86).

#### 2.2.3 Drinking Motivations Questionnaire–Revised (DMQ-R)

In order to assess the motives underlying drinking behavior, the 20-item DMQ-R was used at the beginning of the treatment program (Kuntsche, Knibbe, Gmel, & Engels, 2006; Hungarian version: Németh et al., 2011). The four subscales of the questionnaire (conformity, coping, enhancement, and social motives) provided satisfactory degree of internal consistency in the present sample (Cronbach’s α = 0.79–0.90).

### 2.3 Data analysis

Latent class growth analysis (LCGA) was used to identify subgroups of participants based on the evaluation of psychopathological symptom profiles and change trajectories (Jung & Wickrama, 2008). Average item scores of the BSI subscales assessed at the beginning and at the end of the treatment were specified as continuous indicator variables. According to the LCGA approach, within-class variances were set to zero. Starting with the most parsimonious, one-class solution, models with a growing number of latent classes were assessed during an iterative estimation process. The level of model fit was evaluated based on various indices. The most sufficient model should be characterized with lower rates of Akaike Information Criteria (AIC), Bayesian Information Criteria (BIC), Sample Size Adjusted Bayesian Information Criteria (SSA-BIC), and higher level of entropy. More close fit to the data should be considered in case of a significant result of the Lo-Mendel-Rubin Adjusted Likelihood Ratio Test (LMRT) for a given model compared to the previous model with fewer latent classes. Pairwise missing data handling was used. Therefore, those who only had data at the first measurement point only contributed to the estimation of parameters related to the beginning of the treatment, while parameters related to the end of the treatment were estimated based on only those participants’ responses who had data at the second measurement point (covariance coverage = 69.8–100%).

Next, the identified latent classes were validated by analyzing their relationship with age, gender, family history of substance misuse, previous suicide attempt, psychiatric-, AUD-, or SUD-related pre-care before the treatment program, level of harmful alcohol consumption, drinking motives, and treatment reliability change index (RCI; Jacobson & Truax, 1992). The RCI provides a standardized assessment for each participant as to whether an individual change score is statistically significantly different from a difference that could have occurred due to random measurement error alone. It considers the difference of the post- and pre-treatment score, which is divided by standard error of the differences (Ferguson, Robinson, & Splaine, 2002). It is important to note, that it does not inform whether a statistically significant change was caused by a particular intervention program. The validation analyses were carried out with multinomial logistic regression (R3Step) and the Bolck-Croon-Hagenaars (BCH) method (Asparouhov and Muthen, 2013, Asparouhov and Muthen, 2014). Mplus 8.0 and SPSS Statistics 25.0 statistical software were used to perform the analyses.

## 3 Results

### 3.1 Sample characteristics

Sample characteristics are presented in Table 3. A higher proportion of the participants were male, reported a family history of SUD, and most of the respondents reported a psychiatric-related or AUD-related treatment engagement in their lifetime or shortly before the treatment program. Over two-thirds of the participants successfully completed the eight-week long MM program. Most frequently, the treatment was interrupted because of alcohol consumption during the program, while other participants also reported about treatment-based reasons (e.g. ambivalence towards the aims and assignments of the program) non-treatment-based reasons (e.g. occupational, relationship, or administrative problems), or other or undefined reasons for treatment interruption (e.g. patient did not return to the program after weekend).

**Table 3:** Descriptive characteristics of the sample in terms of socio-demographics, psychiatric anamnesis and treatment completion (N = 303).

Sample characteristics	
Gender N (%)	
Females	123 (40.59%)
Males	180 (59.41%)
Age M (SD)	46.43 (10.32)
Family history of substance misuse N (%)	
Yes	197 (65.02%)
No	105 (34.65%)
Previous suicide attempt N (%)	
Yes	58 (19.14%)
No	244 (80.53%)
Psychiatric-, AUD- or SUD-related treatment involvement history N (%)	
Yes	294 (97.03%)
No	9 (2.97%)
Psychiatric- or AUD-related pre-care within 1 months before the treatment program N (%)	
Yes	248 (81.85%)
No	55 (18.15%)

Treatment completion statistics	

Treatment completion status N (%)	
Successful treatment completion	218 (71.95%)
Interruption of the treatment before completion	85 (28.05%)
Reasons of treatment interruption N (%)	
Alcohol consumption	27 (31.76%)
Treatment-based reasons^1^	20 (23.53%)
Non-treatment-based reasons^2^	18 (21.18%)
Other or undefined reasons^3^	20 (23.53%)

Note. ^1^Treatment-based reasons: ambivalence towards the aims and assignments of the program, non-completion of the assignments of the treatment, violation of treatment rules (e.g., use of mobile phone), acting out, feelings of doubt about the necessity of treatment, unable to work or open up in group psychotherapeutic sessions, reassignment to psychiatric inpatient department because of severe depressive symptoms, unable to continue treatment because of the circumstances in the treatment department. ^2^Non-treatment-based reasons: occupational, relationship, or administrative problems. ^3^Other or undefined reasons: patient did not return to the program after weekend without notification, no available reason of treatment interruption.

### 3.2 Latent class growth analysis (LCGA)

A latent class growth analysis (LCGA) was performed to identify latent classes based on distinct symptomatic profiles and to examine psychopathological symptom change trajectories. Models which contained one to four latent classes were evaluated. Table 4 contains the fit indices for the LCGA models with different number of latent classes. The four-class solution presented the lowest rates of AIC, BIC and SSA-BIC. However, the LMRT showed a non-significant result (*p* > 0.05) for the model with four latent classes. Therefore, the inclusion of an additional subgroup in the model over three latent classes did not contribute to a more optimal degree of model fit. For further analyses the three-class model was retained. In case of the three-class solution, the average latent class probabilities for the most likely latent class membership were 0.94, 0.96, and 0.98, respectively.

**Table 4:** Fit indices for the latent class growth analysis models based on the scales of the Brief Symptom Inventory.

	AIC	BIC	SSA-BIC	Entropy	LMRT	*p*
1-class model	10618.05	10766.34	10639.48			
2-class model	8466.55	8692.68	8499.22	0.955	2175.36	<0.001
3-class model	7811.61	8115.59	7855.53	0.946	691.17	0.016
4-class model	7386.42	7768.26	7441.60	0.935	463.32	0.403

Note. AIC = Akaike Information Criteria; BIC = Bayesian Information Criteria; SSA-BIC = Sample Size Adjusted Bayesian Information Criteria; LMRT = Lo-Mendel-Rubin Adjusted Likelihood Ratio Test.

**Fig. 1:**
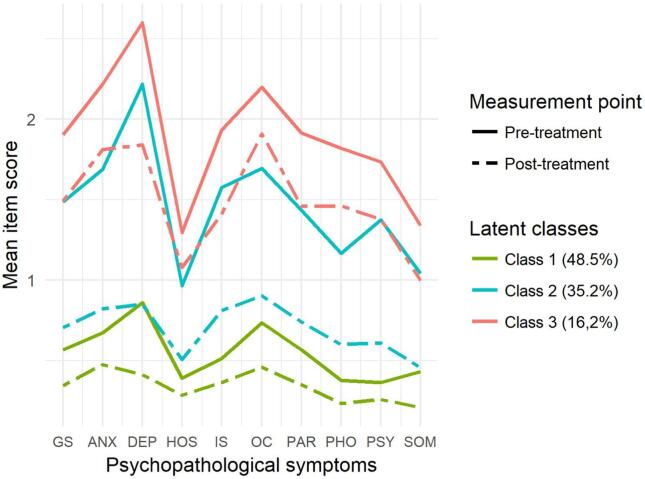
Mean item scores of the three latent classes on the subscales of the BSI before and after the treatment program. Abbreviations: GS = Global symptom severity, ANX = Anxiety; DEP = Depression; HOS = Hostility; IS = Interpersonal sensitivity; OC = Obsessive-compulsive; PAR = Paranoid ideation; PHO = Phobic anxiety; PSY = Psychoticism; SOM = Somatization.

Fig. 1 and Table 5 demonstrate the symptom profiles of the three identified latent classes at the beginning and the end of the treatment program. Apart from the change of hostility for Class 3, latent classes showed significant decreases in each dimension of psychopathological symptoms. Generally, within each latent class, participants experienced anxiety, depression, and obsessive-compulsive symptoms at the highest severity levels. Individuals assigned to Class 1 (“low severity symptomatic subgroup with mild decrease”) had low-severity symptom profiles at both measurement points. For example, at the beginning of the treatment period, a 43-year-old female patient who was a member of this class reported experiencing most frequently psychiatric symptoms “slightly” and “not at all”. At the end of the treatment, this patient was predominantly free of psychiatric symptoms. Individuals assigned to Class 2 (“moderate severity symptomatic subgroup with strong decrease”) had moderate levels of symptomatic severity at the beginning of the treatment, but low levels of symptomatic severity by the end of the program. For example, at the beginning of the treatment period, a 47-year-old male patient who was a member of this class reported experiencing most frequently symptoms of (i) anxiety, interpersonal sensitivity, obsessive-compulsivity, paranoid ideation, psychoticism and somatization “slightly” and “moderately”, (ii) phobic anxiety “moderately” and “fairly”, and (iii) depressive symptoms “fairly” and “extremely”. At the end of the treatment, this patent mostly reported experiencing psychiatric symptoms “slightly” and “not at all”. Individuals assigned to Class 3 (“high severity symptomatic subgroup with moderate decrease”) had high levels of symptomatic severity at the beginning of the treatment program, but moderate levels of symptomatic severity by the end of the program. For example, at the beginning of the treatment period, a 59-year-old female patient who was a member of this class reported experiencing most frequently symptoms of (i) psychoticism “moderately” or “not at all”, (ii) anxiety, depression, hostility, interpersonal sensitivity, paranoid ideation and somatization “moderately” and “fairly”, and (iii) obsessive compulsivity “fairly” and “extremely”. At the end of the treatment this patient most frequently experienced symptoms of (i) hostility and psychoticism “not at all” and slightly”, (ii) anxiety, depression and paranoid ideation “slightly”, (iii) interpersonal sensitivity “slightly” and “moderately”, (iv) phobic anxiety “slightly” and “extremely”, (v) symptoms of obsessive-compulsivity “moderately”, and (vi) symptoms of somatization “fairly”.

**Table 5:** Comparisons of the latent classes in terms of alcohol-related variables.

	Class 1 “Low severity symptomatic subgroups with mild decrease” N = 146; 48.5%	Class 2 “Moderate severity symptomatic subgroup with strong decrease” N = 106; 35.2%	Class 3 “High severity symptomatic subgroup with moderate decrease” N = 49; 16.2%	Overall Wald test (*p*)
*Parameter estimates of LCGA*				
Global symptom severity I (S)	0.57 (−0.22)	1.49 (−0.78)	1.90 (−0.41)	
Anxiety I (S)	0.67 (−0.20)	1.69 (−0.87)	2.22 (−0.41)	
Depression I (S)	0.86 (−0.45)	2.22 (−1.37)	2.60 (−0.76)	
Hostility I (S)	0.39 (−0.10)	0.97 (−0.46)	1.30 (−0.22)	
Interpersonal sensitivity I (S)	0.51 (−0.15)	1.58 (−0.76)	1.93 (−0.52)	
Obsessive compulsive I (S)	0.74 (−0.28)	1.69 (−0.79)	2.20 (−0.29)	
Paranoid ideation I (S)	0.57 (−0.22)	1.43 (−0.69)	1.92 (−0.46)	
Phobic anxiety I (S)	0.38 (−0.14)	1.17 (−0.57)	1.82 (−0.36)	
Psychoticism I (S)	0.37 (−0.11)	1.37 (−0.76)	1.74 (−0.36)	
Somatization I (S)	0.43 (−0.22)	1.04 (−0.59)	1.34 (−0.34)	

*Comparisons*				
Harmful alcohol consumption^1^ M (SE)	−0.27 (0.09)_b_	0.22 (0.09)_a_	0.32 (0.15)_a_	20.93 (<0.001)
Conformity drinking motive^1^ M (SE)	−0.23 (0.08)_b_	0.18 (0.13)_a_	0.34 (0.20)_a_	12.89 (0.002)
Coping drinking motive^1^ M (SE)	−0.40 (0.10)_b_	0.41 (0.09)_a_	0.29 (0.17)_a_	39.37 (<0.001)
Enhancement drinking motive^1^ M (SE)	−0.23 (0.09)_b_	0.17 (0.10)_a_	0.37 (0.21)_a_	12.21 (0.002)
Social drinking motive^1^ M (SE)	−0.18 (0.09)_b_	0.18 (0.11)_a_	0.14 (0.20)_a,b_	6.94 (0.031)
Reliable Change Index (RCI) – Global Symptom Severity^2^ M (SE)	−1.90 (0.29)_a_	−7.73 (0.59)_b_	−2.83 (1.18)_a_	77.13 (<0.001)

Note. The presented parameter estimates of LCGA are intercepts (I) and slopes (S) in brackets. Except the change of Hostility for Class 3 (*p* = 0.177), mean slope estimates in each latent classes were significant at least *p* < 0.05 level. In the case of comparisons, means (standard errors in brackets) in the same row that do not share subscripts differ at *p* < 0.05 level. BCH method was used in the comparison (Asparouhov and Muthén 2014). ^1^Variables measured at pre-treatment and standardized (variables’ mean equals to 0 and standard deviation equals to 1) in order to ease interpreation.^2^Lower values represent more reliable symptom decrease in terms of global psychopathological severity.

### 3.3 Validation of the latent classes

The identified latent classes were contrasted in terms of alcohol consumption-related variables. Table 5 shows the results of the multiple comparisons. The “low severity” subgroup significantly demonstrated the lowest rates on alcohol consumption-related variables at the beginning of the treatment program. The “moderate severity” and “high severity” subgroups significantly demonstrated higher levels of harmful alcohol consumption and drinking motives at the beginning of the treatment program.

Next, multinomial logistic regression analysis was performed to examine the association between latent class membership and psychopathological history-related and alcohol consumption-related covariates (Table 6). The “low severity” subgroup was selected as the reference category. The presence of family history of substance misuse, absence of pre-care before the treatment program, higher rates of coping drinking motives and harmful alcohol consumption all significantly increased the odds of being in the “moderate severity” subgroup membership compared to the reference category. In the case of the “high severity” subgroup, higher rates of conformity and coping drinking motives significantly contributed to the class membership compared to the reference category.

**Table 6:** Odds ratios (95% Confidence Intervals) of the association between validating covariates and latent class membership relative Class 1 (“Low severe symptomatic subgroups with mild decrease”).

	Class 2 “Moderate severity symptomatic subgroup with strong decrease” N = 105; 34.9% OR [95% CI]	Class 3 “High severity symptomatic subgroup with moderate decrease” N = 49; 16.2% OR [95% CI]
Age	1.01 [0.98 – 1.05]	1.02 [0.97 – 1.08]
Gender^1^	1.36 [0.66 – 2.82]	1.99 [0.75 – 5.29]
Family history of substance misuse^2^	**2.13 [1.04 – 4.33]**	2.25 [0.91 – 5.58]
Previous suicide attempt^2^	1.39 [0.60 – 3.19]	1.75 [0.66 – 4.67]
Psychiatric-, AUD- or SUD-related pre-care shortly before the treatment program ^2^	**0.36 [0.14 – 0.90]**	0.35 [0.11 – 1.13]
Harmful alcohol consumption	**1.48 [1.00 – 2.19]**	1.31 [0.72 – 2.38]
Conformity drinking motive	1.50 [0.93 – 2.40]	**1.81 [1.06 – 3.08]**
Coping drinking motive	**2.53 [1.65 – 3.88]**	**1.85 [1.03 – 3.31]**
Enhancement drinking motive	1.10 [0.70 – 1.72]	1.36 [0.64 – 2.91]
Social drinking motive	1.19 [0.74 – 1.92]	1.65 [0.79 – 3.46]

Note. Odds ratios presented by bold figures are significant at least *p* < 0.05 level. ^1^Gender: 0 = Female, 1 = Male; ^2^Categorical variables coded as 0 = No, 1 = Yes.

In terms of the symptom change reliability index, individuals in the “moderate severity” subgroup significantly demonstrated the highest rates of reliable symptom decreases (Table 5, Table 7). Compared with members of the other classes, they showed the highest level of non-random measurement error-based symptom decrease (Table 5), and significantly higher proportion of this class was categorized with reliable symptom decrease (as opposed to non-reliable change or reliable increase of symptoms) compared to the “light severity” class.

**Table 7:** Association between treatment completion status, reliable change index categories and latent class membership.

	Class 1 “Low severity symptomatic subgroups with mild decrease” N = 146; 48.5%	Class 2 “Moderate severity symptomatic subgroup with strong decrease” N = 106; 35.2%	Class 3 “High severity symptomatic subgroup with moderate decrease” N = 49; 16.2%
*Treatment completion status*			
Successfully completed treatment; N = 218 (71.9%)	110 (75.3%)	77 (72.6%)	30 (61.2%)
Dropped from treatment; N = 85 (28.1%)	36 (24.7%)	29 (27.4%)	19 (38.3%)
OR [95% CI]* - Successful completion	*Ref.*	0.87 [0.49–1.54]	0.52 [0.26–1.03]

*Categories of Reliable Change Index (Global symptom severity)*			
Reliable decrease of symptoms N = 134 (63.8%)	50 (47.2%)	66 (88.0%)	18 (62.1%)
Non-reliable change N = 59 (28.1%)	49 (46.2%)	5 (6.7%)	5 (17.2%)
Reliable increase of symptoms N = 17 (8.1%)	7 (6.6%)	4 (5.3%)	6 (20.7%)
OR [95% CI]* - Reliable decrease	*Ref.*	**8.21 [3.71–18.17]**	1.83 [0.79–4.25]

Note. Percentages in each cells represents the proportion within each latent classes. Treatment completion status: χ^2^(2) = 3.66; *p* = 0.160. Categories of Reliable Change Index: χ^2^(4) = 44.05; *p* < 0.001. OR: odds ratio. CI: confidence interval. *Comparison group is Class 1 (Ref. = reference group). A dichotomous outcome variable was constructed for comparisons: 0 = Non-reliable change or reliable increase of symptoms, 1 = Reliable decrease of symptoms. OR presented with bold figures are significant at least *p* < 0.05 level.

In terms of treatment completion, 24.7%, 27.4% and 38.3% treatment attrition rates were presented for the “low severity”, “moderate severity” and “high severity” classes, respectively. There was a non-significant relationship between treatment completion status and latent class membership (Table 7).

## 4 Discussion

The present study aimed to identify subgroups of participants with AUD attending a twelve-step based treatment program based on psychopathological symptom profiles and change trajectories. To the best of the authors’ knowledge, no previous study has examined how latent classes of AUD change during treatment attendance in terms of co-occurring psychopathological symptom levels. Three latent classes were identified: (i) a low severity symptomatic subgroup at baseline with mild decrease, (ii) a moderate severity symptomatic subgroup at baseline with strong decrease, and (iii) a high severity symptomatic subgroup at baseline with moderate decrease.

In line with some of the previous findings, quantitative differences were observed between the subgroups (Urbanoski et al., 2015, Villalobos-Gallegos et al., 2017). Namely, classes were separated by symptom profiles with intensifying severity at both measurement points. However, it is important to note, the present study had limited assessment of externalizing characteristics (e.g., absence of antisocial personality disorder, drug misuse, etc.) which might have influenced characteristics of the latent classes. This data pattern also corresponds with the concept of hierarchical structure of psychopathology: a higher order dimension of internalizing psychopathology might explain the interrelations of psychopathological symptoms within each class and represent a severity-based risk for experienced psychopathological difficulties (Kotov et al., 2017, Villalobos-Gallegos et al., 2017).

The identified latent class model is also comparable with previous studies using latent class analysis in treatment seeking or general population samples of individuals with AUD. The “low severity” subgroup had comparable symptom profile to the “low comorbidity” AUD subtype by Müller et al. (2019), the “mild” class by Villalobos-Gallegos et al. (2017) or the “comorbidity unaffected” group by Glass et al. (2014). The “moderate severity” subgroup corresponded with the “moderate” class by Villalobos-Gallegos et al. (2017). Finally, the “high severity” subgroup presented similar characteristics to the “internalizing comorbidity” group by Glass et al. (2014), “multimorbidity” class by Urbanoski et al. (2015) or the “moderate/severe” class by Villalobos-Gallegos et al. (2017).

The subgroups not only differed by severity of symptoms, but also showed different levels of symptomatic change during the eight-week long period. Symptom decrease with the largest and most reliable (non-measurement error-related) effect was demonstrated in the case of the “moderate severity” subgroup. The “low severity” and “high severity” subgroups demonstrated significant but less intensive attenuation in each of the psychopathological domains. However, it is important to highlight, that design of the present study (e.g., lack of randomized controlled trial) was unable to determine whether these patterns of symptomatic decrease were attributable to the effect of the Minnesota Model treatment. Therefore, it limits the possibility of linking the present findings to previous twelve-step based treatment related research data which has demonstrated that attendance in these interventions can lead to attenuation of psychopathological symptoms (e.g., depression symptoms; Worley et al., 2012). However, by using a latent class-based approach it was possible to examine more specifically if different subgroups of AUD demonstrated different trajectories in terms of psychopathological symptom reductions (Lanza & Rhoades, 2013).

The present study also explored the association between latent class membership and alcohol consumption-related variables. Participants in the moderate and high severity symptomatic subgroups presented significantly higher rates of baseline harmful alcohol consumption. These findings are consistent with previous empirical research which have demonstrated that some subgroups of AUD with increased severity of alcohol misuse are also characterized with more serious internalizing and externalizing symptoms (Hildebrandt et al., 2017, Moss et al., 2010). Additionally, multivariate analyses identified the substantial role of baseline coping and conformity motives in the cases of the more severely affected classes. Both coping and conformity motives have been described as negative reinforcement-based motives of drinking which are implicated in self-medication tendencies of the participants. Regarding the coping motives, it was assumed that that alcohol consumption serves as a form of emotion regulation among patients with a higher severity symptomatic level, which helps individuals mitigate and cope with unpleasant feelings and emotions (Berking et al., 2011). Previous studies have also demonstrated that subtypes of AUD with elevated internalizing symptomatology show increased rates of drinking in order to relief or self-medicate psychological distress (Hildebrandt et al., 2017, Müller et al., 2019). In the case of conformity motives, it was hypothesized that the “high severity” symptomatic class might show tendencies also to use alcohol as a means for reducing symptoms related to social anxiety (Villarosa, Madson, Zeigler-Hill, Noble, & Mohn, 2014).

### 4.1 Limitations

The present findings should be interpreted cautiously due to several limitations related to the study. First, the lack of control comparison group impeded to accurately interpret the efficacy of the MM in terms of psychopathological symptom reduction. Second, due to the absence of follow-up data collection, the present design did not assess the long-term alcohol use-related and psychopathological-related outcomes among the participants who successfully completed the program and among those who dropped out from it. Third, the present study did not examine the role of potential third variables which might have mediated or moderated the treatment effect (e.g., AA involvement, comorbid psychiatric diagnosis). Fourth, it is important to consider that the composition of the present sample was based on availability of the patients, therefore the generalizability of the results to a broader population with AUD is arguably limited. For example, findings from the National Epidemiologic Survey on Alcohol and Related Conditions (NESARC) and a nationally representative sample from Hungary have both shown that the lifetime treatment rates for AUD among those with AUD and in the latent class of ‘Alcohol drinkers with severe dependence symptoms’ are much lower than those in the present sample (Hasin and Grant, 2015, Horváth et al., 2019). As the applied treatment form was highly-structured and built upon the principles of the twelve-step approach, it might contribute to a self-selection effect of more motivated patients with a history of psychiatric treatment involvement. Fifth, as the research assessment was conducted by the treatment staff, it might had some effect on the participants’ response tendencies, in addition to the possible bias in responses due to the participation of affected family members in the admission interview. Sixth, design of the present study did not allow to analyze the causal relationship between AUD and psychopathological symptoms. Finally, the effects of important covariates were not controlled during the analyses which might have influenced profile characteristics and changed trajectories of psychopathological symptoms, such as effects of detoxification in the first weeks of the treatment and potential period effects related to changes in therapeutic characteristics over the five-year period of the study.

## 5 Conclusions

The present study examined psychopathological symptom profiles and change trajectories among patients undergoing a twelve-step based MM treatment. The present study identified three severity-based subgroups of inpatients with AUD undergoing MM treatment. During the eight-week long period of the study, each of the three AUD severity classes demonstrated significant reductions in terms of psychopathological symptoms. Further studies, with more precise methodological design, are warranted to provide evidence whether structured, more intensive, and community-based residential treatment forms that facilitate twelve-step involvement can contribute to beneficial outcomes among AUD patients with more severe psychopathological symptomatic profiles (Karriker-Jaffe, Klinger, Witbrodt, & Kaskutas, 2018). Previous studies have suggested that integrated treatment forms, which simultaneously address AUD-related and psychological-related impairments might have beneficial effects among patients with comorbid AUD and psychiatric disorders (McClean, Anspikian, Winters, & Tsuang, 2014). Interventions which combined treatment approaches focusing on AUD and co-occurring psychiatric disorders were used to facilitate simultaneous improvements in symptomatology of AUD as well as comorbid disorders. For example, in the cases of comorbid AUD and internalizing psychiatric disorders, one might consider teaching effective coping and emotion-regulation strategies to control negative emotions. This includes cognitive restructuring techniques to explore and correct situational and cognitive risk processes (e.g., beliefs) underlying AUD and comorbid internalizing disorders (e.g., anxiety, depression disorders), and understanding and altering expectancies and motivational processes of alcohol use which can be associated with symptoms of negative affectivity (Morris et al., 2005, Roberts et al., 2015).

## Role of funding sources

The study was supported by the Hungarian National Research, Development and Innovation Office (Grant numbers: K111740, KKP126835, NKFIH-1157-8/2019-DT). Judit Farkas was supported by the 10.13039/501100005881ÚNKP-17-4 of the new National Excellence Program of the Hungarian Ministry of Human Capacities.

## Contributors

Zsolt Horváth, Mark D. Griffiths, Zsolt Demetrovics and Róbert Urbán wrote the manuscript. Mariann Tremkó, Zsolt Fazekas, András Tóth, Zsolt Petke and Judit Farkas designed the study and performed data collection. Zsolt Horváth conducted statistical analyses under Róbert Urbán’s supervision. All authors have critically revised the manuscript and approved its final version.

## Declaration of Competing Interest

The authors declare that they have no known competing financial interests or personal relationships that could have appeared to influence the work reported in this paper.
